# Small Intestine Ectopic Varices as a Cause of Obscure Gastrointestinal Bleeding in a Cirrhotic Patient

**DOI:** 10.7759/cureus.65959

**Published:** 2024-08-01

**Authors:** Hazem Abosheaishaa, Omar Abdelhalim, Yasser Hegazy, Abdelrahman Abdelwahed, Nourhan Ahmed, Mahmoud Nassar

**Affiliations:** 1 Internal Medicine, Icahn School of Medicine at Mount Sinai, Queens Hospital Center, New York, USA; 2 Internal Medicine, East Carolina University, Greenville, USA; 3 Internal Medicine, Faculty of Medicine Cairo University, Cairo, EGY; 4 Endocrinology, Diabetes and Metabolism, Jacobs School of Medicine and Biomedical Sciences, University of Buffalo, Buffalo, USA

**Keywords:** endoscopy, histoacryl injection, ectopic varices, liver cirrhosis, portal hypertension, gastrointestinal bleeding

## Abstract

Portal hypertension is a major complication of liver cirrhosis, leading to various life-threatening conditions. The most common of these is the formation and bleeding of varices at the portosystemic anastomosis. Varices are most commonly esophageal or gastric and less commonly ectopic. Although ectopic varices are rare, they should be considered as a cause of obscure gastrointestinal bleeding in cirrhotic patients. We present a case of ruptured ectopic varices in the small intestine of a known cirrhotic patient who presented with anemia and melena, alternated with hematochezia. The case was managed with Histoacryl^®^ injection using push enteroscopy, resulting in adequate hemostasis.

## Introduction

Liver cirrhosis is an end-stage liver condition caused by long-term liver damage resulting from various chronic liver diseases, both infectious and non-infectious. These include alcoholic liver disease, autoimmune liver disease, metabolic diseases (such as MASLD or metabolic dysfunction-associated steatotic liver disease, Wilson's disease, and hemochromatosis), and hereditary abnormalities like alpha 1-antitrypsin deficiency. Liver cirrhosis is the sixth leading cause of adult mortality. The most severe prognostic factor of cirrhosis is the presence of portal hypertension (PHT), which increases the risk of secondary complications such as encephalopathy, ascites, variceal bleeding, and hepatocellular carcinoma [1-4].

Rupture of esophageal and gastric varices is the most common cause of gastrointestinal (GI) bleeding. However, the rarely occurring ectopic varices (EcV) can be a cause of obscure GI bleeding. EcVs are dilated venous portosystemic collaterals that appear in unexpected locations other than the usual gastroesophageal area. This term refers to any abnormally dilated tortuous veins connected with the gastrointestinal mucosa, as well as any portosystemic collateral veins seen in a PHT patient's abdominal wall or retroperitoneum. Because EcVs are uncommon, most of the existing data on them, including locations, frequencies, and intervention techniques, is based on case reports and small case series. Raising awareness of EcV is critical for gastroenterologists managing GI bleeding, not only because they account for 1-5% of all variceal bleeds in patients with intrahepatic PHT and 20-30% of those with extrahepatic PHT but also because of the difficulty in management and the substantial mortality rate associated with their initial bleeding (up to 40%) [5,6].

## Case presentation

A 67-year-old male patient known to be diabetic on insulin, hypertensive on calcium channel blockers (CCB) and angiotensin receptor blockers (ARBS), with liver cirrhosis secondary to chronic hepatitis C. He received direct antiviral agents (DAAs) for HCV (sofosbuvir 400 mg and daclatasvir 60 mg) for 12 weeks with sustained viral response (SVR). He was admitted to the ICU with encephalopathy, recurrent melena, and hematochezia. Upon admission, his vital data were as follows: BP: 100/60 mmHg, Pulse: 112/ min, respiratory rate (RR): 16/min, and temperature: 37 °C. On general examination, the patient was drowsy, disoriented, and agitated. The conjunctiva showed severe pallor and the extremities showed mild lower limb edema. On local examination, the patient showed hepatosplenomegaly with mild distention in the abdomen without tenderness or rigidity. The review of other systems was unremarkable.

**Figure 1:**
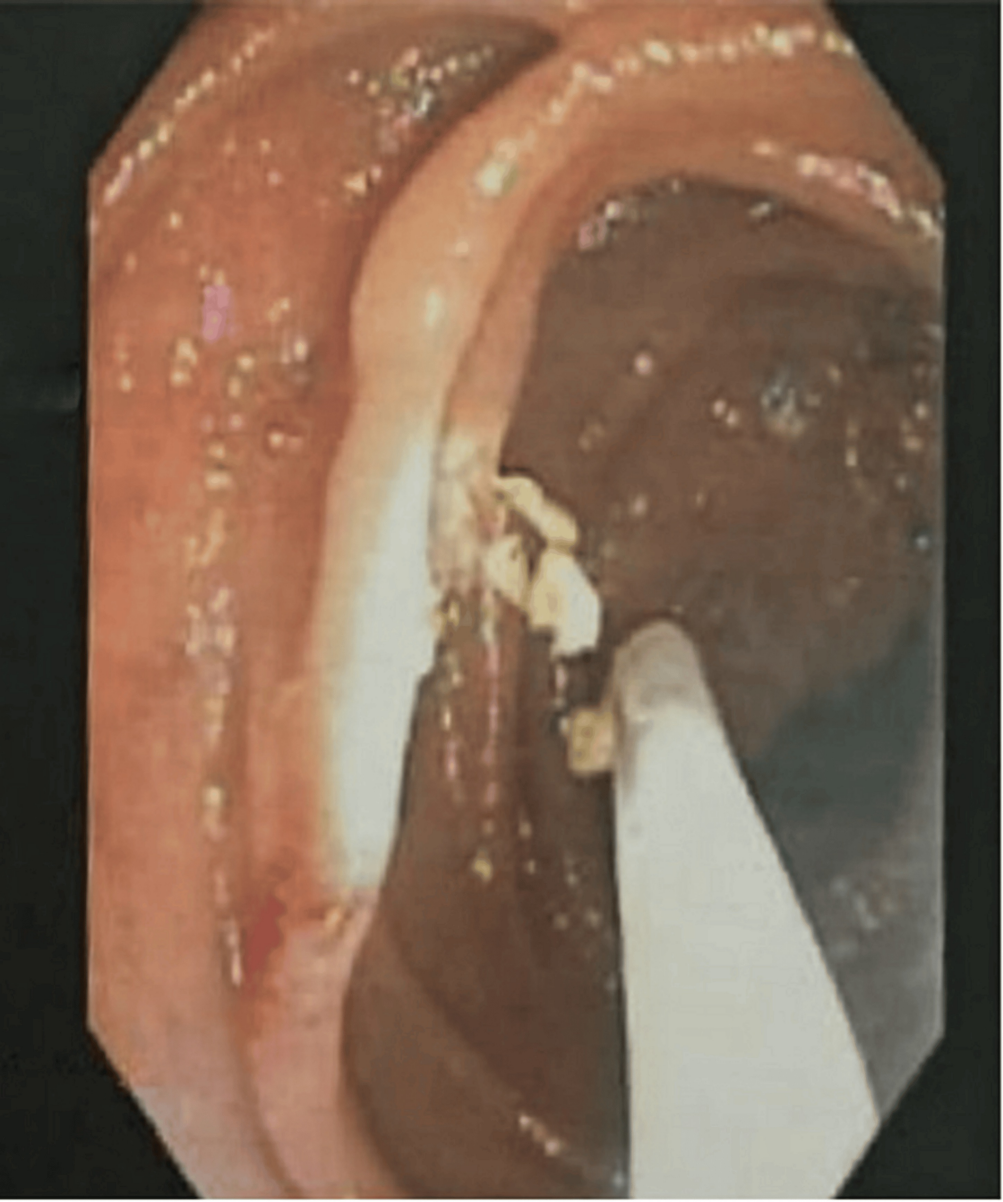
Histoacryl injection with successful hemostasis

Initial laboratory data were as follows: WBC: 10,600/mcL, hemoglobin: 6.5 g/dl, platelet count 53000/mcL, total bilirubin 1.87 mg/dL, serum albumin 2.8 mg/dl, and INR was 1.65. The patient was resuscitated with four packed RBCs and IV crystalloids and treated with proton-pump inhibitors (PPIs), and terlipressin. After stabilization, the upper GI endoscopy was done and revealed grade I varices with mild portal hypertensive gastropathy with no bleeding stigmata. Then, a colonoscopy with done to find out the source of the bleeding, and it was normal. Upon continuous bleeding, a small intestine push enteroscopy was done and revealed a jejunal ectopic varix with stigmata of recent bleeding. Histoacryl^®^ injection was given and successful homeostasis was achieved (Figure 1). Following the procedure, the patient was transferred to a ward in stable condition. A follow-up laboratory investigation was done after two days with no hemoglobin drop. After the stoppage of melena, the patient was discharged and attended the gastroenterology clinic two weeks later in stable condition with no bleeding recurrence.

## Discussion

Being relatively uncommon, data about ectopic varices are available only from case reports and some small case series. Norton ID et al. were the only ones to analyze a significant case series (169) of bleeding from ectopic varices [7]. Twenty-six of those were peristomal in source, 17% in the duodenum, 17% in the jejunum or ileum, 14% in the colon, 8% in the rectum, 9% in the peritoneum, and a few in other uncommon locations such as the vagina and ovary. EcV is more commonly encountered in clinical practice nowadays because of the substantial advancement in diagnostic methods like double-balloon enteroscopy and capsule endoscopy [8]. In reality, approximately 8.1% of individuals with PHT who underwent capsule endoscopy have small intestinal varices [9]. Limited data make the intervention for bleeding ectopic varices a big challenge, as it requires proper determination of the site of bleeding, studying the vascular supply, choosing the appropriate method to control bleeding in a well-prepared hospital with well-trained staff, including gastroenterologist, hepatologist, intervention radiologist, and GI surgeons [10]. Akhter and Haskal et al. provided a thorough review of existing modalities of therapy and the latest developments in managing ectopic variceal bleeding, including double balloon enterostomy and transcatheter embolization or injection sclerotherapy, with or without portosystemic decompression, also known as transjugular intrahepatic portosystemic shunts (TIPS) [11]. Furthermore, different modalities used outside of the acute setting have shown to be effective adjuncts. Push enteroscopy can transverse the small bowel and allow for intervention, as in our instance. Duodenal and colonic varices have also been effectively diagnosed using capsule endoscopy, CT, CT angiography, and CT enteroclysis [12-14]. The significance of TIPS in the treatment of bleeding ectopic varices in cirrhotic patients caused by intrahepatic portal hypertension is generally recognized [15].

Ultimately, because liver disease patients have a high risk of morbidity and death, surgical intervention is still the last resort. However, it may be deemed as the last resort option in a small subset of patients whose other treatment options have failed.

The diagnosis and management of ectopic varices, particularly in patients with portal hypertension, remain complex and challenging due to their rarity and varied anatomical locations. Advanced diagnostic techniques have significantly improved the detection of these varices. Double-balloon enteroscopy, for instance, allows for detailed visualization and therapeutic intervention within the small intestine, enabling the identification and treatment of obscure sources of gastrointestinal bleeding. Capsule endoscopy has also become a valuable tool in the non-invasive diagnosis of small bowel varices, providing a comprehensive view of the gastrointestinal tract that can detect bleeding sources missed by other modalities. In cases where bleeding ectopic varices are identified, endoscopic interventions, such as band ligation or sclerotherapy, can be employed, although these are often challenging due to the anatomical complexity of the varices [16-18].

Moreover, the role of TIPS in managing bleeding from ectopic varices has been well-documented. TIPS helps reduce portal pressure, thereby decreasing the likelihood of variceal bleeding. It is particularly beneficial for patients who are not candidates for other endoscopic or surgical interventions. Despite these advancements, the prognosis for patients with bleeding ectopic varices remains guarded, and multidisciplinary care involving gastroenterologists, hepatologists, interventional radiologists, and surgeons is crucial for optimal outcomes. Research continues to explore new therapeutic options and improve existing techniques to enhance the management of this challenging condition [19,20].

## Conclusions

Ectopic varices present a significant clinical challenge in both diagnosis and management due to their uncommon locations outside the esophagus and stomach. Current management strategies are primarily based on expert experience rather than robust clinical trials, leading to variability in treatment approaches. This underscores the urgent need for higher-level evidence research to develop standardized guidelines and improve patient outcomes. Enhanced diagnostic techniques and innovative therapeutic strategies are essential to address the complexities associated with ectopic varices effectively.
